# Downmodulation of Regulatory T Cells Producing TGF-β Participates in Pathogenesis of Leprosy Reactions

**DOI:** 10.3389/fmed.2022.865330

**Published:** 2022-07-18

**Authors:** Katherine Kelda Gomes de Castro, Pedro Henrique Lopes da Silva, Luciana Nahar dos Santos, Julia Monteiro Pereira Leal, Mylena Masseno de Pinho Pereira, Iris Maria Peixoto Alvim, Danuza Esquenazi

**Affiliations:** ^1^Leprosy Laboratory, Oswaldo Cruz Institute, Oswaldo Cruz Foundation, Rio de Janeiro, Brazil; ^2^Department of Pathology and Laboratories, School of Medical Sciences, State University of Rio de Janeiro, Rio de Janeiro, Brazil

**Keywords:** leprosy, reactions, T lymphocytes, Treg, cytokines

## Abstract

Leprosy reactions are an acute and systemic manifestation, which occurs suddenly, can be severe and lead leprosy patients to disability. Reactional episodes are observed among half of the multibacillary patients, mainly in borderline lepromatous and lepromatous forms. They may begin at any time during multidrug therapy, and even before the treatment. Physical disabilities, which are the source of extreme suffering and pain for patients, occur in progression of the cellular immune response associated with a reaction and are still poorly understood. Thus, this work aimed to phenotypically and functionally characterize CD4^+^ and CD8^+^ Treg cells *ex vivo* and in response to *Mycobacterium leprae* (ML). We studied 52 individuals, including 18 newly diagnosed and untreated multibacillary leprosy patients, 19 reactional multibacillary patients (Type I or Type II episodes) and 15 healthy volunteers, included as controls, all residents of the city of Rio de Janeiro. The functional activity and frequencies of these cells were evaluated through multiparametric flow cytometry. In addition, the production of cytokines in supernatant from peripheral blood mononuclear cell cultures was also investigated against ML by enzyme-linked immunosorbent assay. Our results showed a decrease in CD4^+^TGF-β^+^ Treg and CD8^+^ TGF-β^+^ Treg in leprosy multibacillary patients during both types of reactional episodes. Alterations in the cytokine profile was also observed in Type II reactions, along with upregulation of IL-17 and IL-6 in supernatant. Thus, our study suggests that downregulation of Treg cells is related with both classes of reactional episodes, improving our understanding of immune hyporesponsiveness in multibacillary patients and hyperesponsiveness in both reactions.

## Introduction

Leprosy is a chronic systemic infectious disease that affected 202,256 new individuals worldwide in 2019 (1). Brazil occupies the second place in newly diagnosed cases, totaling 27,863 patients (1). The etiological agent, *Mycobacterium leprae*, mainly infects macrophages, Schwann cells and the vascular endothelium (2). The disease causes lesions in skin and peripheral nerves, promoting loss of sensitivity, deformities in the limbs/face and even blindness (3). Leprosy presentation differs according to the host immune response to the pathogen, which leads to a clinical spectrum of polar clinical forms, which varies from the tuberculoid (TT) to lepromatous (LL) pole, including the intermediate forms called borderline (BT, BB and BL) and the indeterminate form (I) (4, 5).

Leprosy reactions are the main cause of morbidity and permanent damage to peripheral nerves. About 50% of leprosy patients develop this clinical condition, which is an acute inflammatory response manifested with local and systemic involvement (6, 7). These episodes can manifest at any time throughout the course of the disease, may affect patients of all clinical forms (6), and are classified into Type 1 (T1R) and Type 2 (T2R) reactions (8). It is not yet clear whether changes in immune response patterns are associated with the pathogenesis of reactions.

Borderline patients are the most affected by T1R, which develop gradually and may have a natural course of several weeks. Clinically, in this reaction, there is an increase in the inflammatory process in existing lesions, as well as the appearance of new skin lesions and painful neuritis (9). The T2R consists of a systemic episode of acute inflammation that occurs primarily in LL and in BL patients. This reaction could occur before, during and after multidrug therapy (MDT). In these episodes, patients develop an acute systemic inflammatory response, have deep and painful subcutaneous nodules, often present high fever, oedema and cyanosis of the extremities, weight loss, general malaise, and damage to nerves, skin, eyes and testicles (3, 10). Several studies aim to investigate immunological mechanisms of susceptibility and identify biomarkers, which can explain, predict and control T1R and T2R reactions (11–15). Activation of T helper 1 (Th1) cells and production of proinflammatory cytokines, such as IFN-γ and TNF, are described by several studies to explain the pathogenesis of T1R and T2R (16–18). Although multibacillary patients are hyporesponsive to *M. leprae*, during onset of reactions they develop a sudden exacerbated immune response against the pathogen (15). One of the most accepted hypotheses regarding the triggering of reactions is a change in the regulatory T cell profile (19).

Regulatory T lymphocytes (Treg) present the CD25^+^FOXP3^+^ profile (20), a cell subset which is important for immune system effector suppression mechanisms and is fundamental in the control of autoimmunity in peripheral organs (22). It has been shown that some microorganisms evade elimination promoted by the immune response through the modulation of regulatory T cell mechanisms (23). Treg can suppress Th1 and Th17 cells through TGF-β and IL-10 production and constitutive molecules, such as CTLA-4 (21, 24).

However, the exact role of Treg during reaction episodes is poorly studied and still very controversial. Azevedo and coworkers observed reduced Treg markers in skin lesion samples of T1R and T2R. This data demonstrates that an abrupt reduction in the frequency of Treg cells can contribute to the trigger of exacerbated inflammatory responses during leprosy reactions (25). Similar results have also been described in other diseases, such as atopic dermatitis (26), active tuberculosis (27), and sepsis (28).

Palermo and coworkers were pioneers in the study of Treg in leprosy. They suggested that these cells play a central role in the *M. leprae*-host interaction. The authors demonstrated a decreased frequency of CD4^+^ Treg in peripheral blood and cutaneous lesions of lepromatous in comparison to tuberculoid patients (29). Others also revelated a higher CD4^+^ Treg in LL patients in comparison to BT/TT (30, 31). Analyzing, CD3^+^CD4^+^ T cells in blood, Saini and collaborators saw lower mean fluorescence intensity (MFI) of FOXP3 and TGF-β cells in T1R and T2R compared to BT and LL patients, respectively (32). In addition, depletion of CD4^+^ Treg participates in T2R pathogenesis and prednisolone treatment upregulates this cell profile in blood leucocytes (33).

Most studies on Treg in leprosy reactions focus on the CD4^+^ subset, and information concerning CD8^+^ cells still very scarce. Previous studies in leprosy, related a higher frequency of CD8^+^ Treg in blood of LL/BL patients in comparison to BT/TT (19, 30). Negera et al. (33) did not observe a difference in CD8^+^ Treg frequency between LL patients with or without T2R, and the same cell profile appears to be depleted during prednisolone treatment but did not increase after treatment (33).

We hypothesized that at some point in the clinical course of leprosy *per se*, Treg downregulation occurs, and this change may trigger reactions in the host. Therefore, the aim of our study was to investigate the participation of CD4^+^ and CD8^+^ Treg in the genesis of both Type 1 and Type 2 reactions through peripheral blood mononuclear cells (PBMC) by flow cytometry. In this context, our results show the involvement of TGF-β producing CD4^+^ and CD8^+^ Treg in maintenance of the hyporesponsive response in multibacillary leprosy patients favoring *Mycobacterium leprae* survival.

## Materials and Methods

### Ethical Considerations

The study was approved by the Institutional Ethics Committee of the Oswaldo Cruz Foundation/FIOCRUZ (protocol number 518/09). All participants were informed, and written consent was obtained prior to specimen collection. For the sake of privacy and wellbeing of the studied individuals, we refrained from disclosing their identity.

### Studied Population

Thirty-seven leprosy patients were selected from both sexes, defined according to clinical profile, and distributed into newly diagnosed and untreated lepromatous (LL) and borderline lepromatous (BL) patients, while T1R and T2R patient samples were collected after the diagnosis of any reactional episode and before specific treatment for the reactions. All individuals were followed up at the Leprosy Unit/Fiocruz. The diagnosis of disease and reactional episodes was based on clinical signs and symptoms presented by the patients, confirmed by the histopathological analysis of skin lesion fragments, according to Ridley and Jopling criteria (4) and the treatment of all patients followed the rules defined by the WHO (34). During the study, any multibacillary patients developed reactional episodes.

Furthermore, 15 healthy donors were included in the study. All individuals lived in Brazil, a leprosy endemic area. Patients with a history of BCG vaccination within the last 3 months, patients under 15, co-infections (HIV, TB, Hepatitis B and C), autoimmune or allergic diseases, diabetes, hypertension and pregnancy were excluded from the study.

### Isolation of Peripheral Blood Mononuclear Cells (PBMC) and *in vitro* Assay

About 40 mL of blood were collected under endotoxin-free conditions from heparinized venous blood of all individuals, and PBMC was isolated by Ficoll-Hypaque (GE Healthcare AB, Uppsala, Sweden) centrifugation. After three washes with sterile phosphate-buffered saline (PBS; Gibco Invitrogen Co., USA) the pellet of cells was resuspended in 5 mL AIM-V medium (Thermo Fisher Scientific, USA). Cell viability was determined by 0.4% sterile Trypan Blue solution (Sigma Aldrich, MO, USA) and ranged from 94 to 98%. PBMCs were adjusted to 1 × 10^6^ (for cytometric analysis) and transferred to U-bottomed 96-well polyethylene plates (Nunc, USA) with 200 μL per well for *ex vivo* and *in vitro* assay evaluation. For *in vitro* assay, cells were added into wells with specific antigen or mitogen, namely: *M leprae* antigen (20 μg/mL) irradiated and sonicated, from armadillo (provided by NIH/ NIAID N01 Al-25469 contract with Colorado State University, CO, USA) and as mitogen Phytohemagglutinin (Sigma, USA-1 μg/mL). In all wells, including unstimulated cultures, 1μg/mL anti-CD28 and anti-CD49d was added (Biolegend, USA). PBMC were maintained for 72 h in AIM-V medium at 37°C with 5% CO_2_. After 68 h of culture, intracellular protein transport blocker (BD GolgiPlugTM, USA−10 μg/mL) was added in order to evaluate the production of cytokines and/or molecules inside the cells. Cells were then stained with specific monoclonal antibodies and analyzed by multiparametric flow cytometry. Response kinetics to *M. leprae* and PHA were previously performed in healthy volunteers (HVs), reaching a peak in cultures at 72 h.

### Flow Cytometry Analysis of Cell Surface and Intracellular Molecules

**Figure 1:**
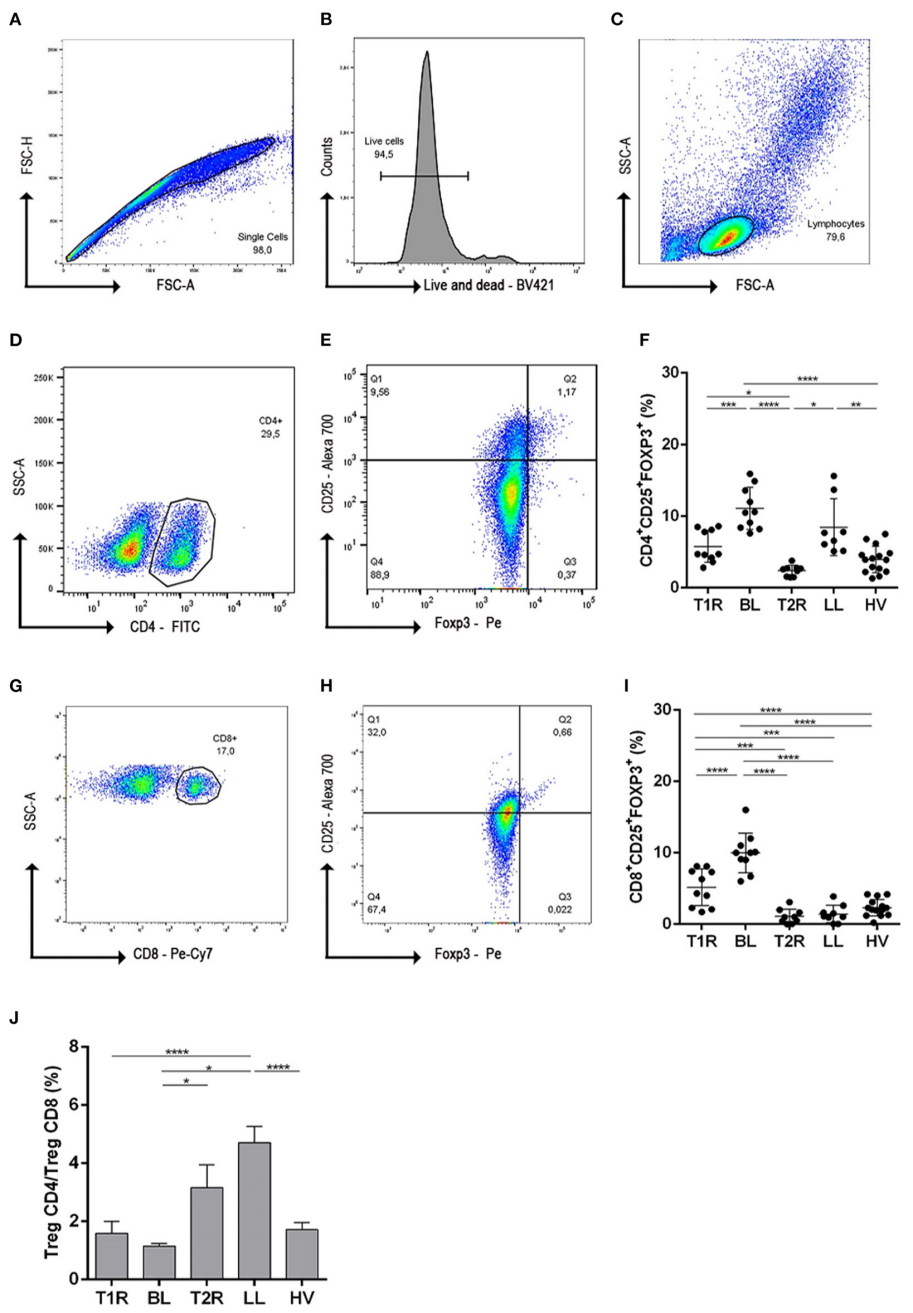
Gating strategies and frequency evaluation of regulatory T cells subsets. To exclude cell doublets and debris from data, cells were gated on singlet regions through dot plot FSC-H vs FSC-A **(A)**; Next analysis was initiated from singlets and gated in live and dead histogram, for viability evaluation **(B)**; lymphocyte population were determined from viable cells in SSC-A vs FSC-A dot-plot **(C)**; the dot-plot from lymphocyte cells was used for CD4^+^ T cell determination **(D)**; CD25 vs Foxp3 gated on CD4^+^ was used to define CD4^+^ T reg cells **(E)**. Graphical representation of CD4+CD25+Foxp3+ frequencies in different clinical forms of leprosy **(F)**. From lymphocyte dot-plot, CD8^+^ cells were gated **(G)** and CD25 vs Foxp3 were defined **(H)** graphical representing of CD8+CD25+Foxp3+ cells **(I)**; Ratio determination of CD4+CD25+Foxp3/CD8+CD25+Foxp3+ frequencies in PBMC **(J)**. In **(F,I)** each symbol represents an individual studied according to the group; horizontal lines represent the mean. In **(J)** each bar represents the mean and standard deviation of the groups and the culture condition. One-way Anova was used for statistical analysis where, *p ≤ 0.05, **p *leq* 0.01, ***p ≤ 0.001 and ****p ≤ 0.0001. T1R, Type 1 Reaction; BL, borderline lepromatous; T2R, Type 2 Reaction; LL, polar lepromatous patients; HV, healthy volunteers.

Following incubation, cells were centrifuged for 5 min at 1,500 rpm at 4°C, at which time supernatants were collected and stored in a −80°C freezer. PBMC were washed twice with PBS (Gibco Invitrogen Co., USA) at 1,500 rpm for 5 min at 4°C. Cells were resuspended in PBS containing 0.02% ethylenediaminetetraacetic acid (EDTA; Sigma), and incubated for 15 min at room temperature, followed by a further wash (1,500 rpm for 5 min at 4°C). PBMCs were stained at a 1:5 ratio with the Live/Dead Kit (Invitrogen, USA) according to manufacturer's instructions. Human TruStain FcXTM—Fc Receptor Blocking Solution (5 μL, Biolegend, USA) was add in 100 μL of PBS/FACS and cells were incubated for 10 min at 4°C. After this step, PBMCs were labeled with different specific monoclonal antibodies (anti-CD4 FITC, anti-CD8 Pe-Cy7, anti-CD25 Alexa 700—Biolegend, USA) and incubated with appropriate isotype controls, used to define the phenotypic subset profiles, for 30 min at 4°C (Biolegend, USA). PBMCs were then resuspended in 1% paraformaldehyde (PA; Sigma) and incubated for 30 min at 4°C. After this step, cells were resuspended in 1% Foxp3 Fix/Perm (Biolegend, USA) and incubated for 20 min at 4°C. PBMCs were subjected to two washes, the first with PBS/FACS and the second with Foxp3 Perm Buffer (1%, Biolegend, USA) and centrifuged at 1,500 rpm for 5 min at 4°C each. Perm Buffer solution (150 μL) was added to PBMCs, which were incubated at room temperature for 15 min and protected from light. Subsequently, PBMCs were stained with monoclonal antibodies for intracellular proteins (anti-FOXP3 Pe, anti-IL-10 APC and anti-TGF-β PerCp—Biolegend, USA) and their respective isotype controls (Biolegend, USA) for 30 min at 4°C. Paraformaldehyde 1% (PA; Sigma) was added and cells were evaluated on the CytoFLEX flow cytometer (Beckamn Coulter, USA). A total of 100,000 events in the strategic lymphocyte region were observed, followed by analysis in FlowJo version 3.0 software (Tree Star Inc., USA) (Figures 1A–E,G,H).

### Cytokine Evaluation by Enzyme-Linked Immunosorbent Assay (ELISA)

ELISA was performed according to the manufacturer's instructions for each kit. Cytokine profile was measured in duplicate using culture supernatants for analysis of IFN-γ, IL-17, IL-23, IL-6, and IL-10 (eBioscience, USA). Supernatants were incubated in 96-well plates (Nunc, Rochester, NY, USA) for 12 h at 4°C with diluted capture antibody for each cytokine. After the sensibilization step, supernatants were washed 3 times with PBS 0.05% Tween 20 after which blocking solution was added to samples that were incubated for 1 h at room temperature. Subsequently, diluted supernatant/standard were added, and plates were incubated overnight at 4°C. Supernatants were washed (three times), diluted detection antibody was added and maintained for 1 h. Avidin-HRP complex (ThermoFisher Scientific, Massachusetts, EUA) was incubated for 30 min at room temperature in the dark and the supernatants were washed (four times). After adding TMB solution (ThermoFisher Scientific, Massachusetts, EUA), the plate was incubated for ~15 min and stop solution was added (2N H_2_SO_4_). Test was performed using SpectraMax 190 (Molecular Devices—USA/Canada) at 450 nm using SoftMax Version 5.3 software (Molecular Devices-US/Canada).

### Statistical Analysis

Before assays, data was tested for normality (Shapiro-Wilk normality test), and non-normally distributed results were analyzed by non-parametric tests. Results were expressed as mean and standard deviation. Significant differences between groups were determined by the non-parametric One Way Anova and Mann Whitney test. GraphPrism version 6.0 (Graph Prism, USA) was used for statistical and graphical analysis. The values with ^*^p ≤ 0.05, ^**^p ≤ 0.01, ^***^p ≤ 0.001 and ^****^p ≤ 0.0001 were considered significant.

## Results

### Clinical and Demographic Characteristics of Volunteers

The participants evaluated in this study were defined according to the clinical presentation of leprosy and monitored at the Leprosy Unit (LAHAN-IOC-Fiocruz). The collection of biological samples occurs after confirmation of the diagnosis of disease and/or reaction episode, immediately before starting MDT or use of anti-reaction drugs (Predinisone and/or Thalidomide). Disease diagnosis was performed by specialized physicians from the Leprosy Unit based on the clinical signs and symptoms presented by patients and confirmed by pathologists through histopathological skin lesion analysis obtained from skin biopsy.

The mean age observed in all groups were similarly around 47 years and the majority of participants were male, accounting for 72%. Bacilloscopic index (BI) mean was 3.9 ± 0.6 in BL patients and 3.5 ± 1.0 in T1R patients (Table 1). Also, the BI mean was 4.6 ± 1.0 in LL patients and 3.4 ± 2.0 in T2R patients. The bacteriological lesion index (BLI) was also analyzed revealing a decreased index in both reactional groups when compared to multibacillary patients (T1R/BL *p* = 0.0449; T2R/LL *p* = 0.0011). Disability grade (DG) was 1 in most reactional patients, while in non-reactional patients the DG was 0. All leprosy patients presented a negative lepromin test. Multibacillary patients presented reactions at different times after starting MDT, 7 ± 1.5 months for T1R and 17.6 ± 12.5 months for T2R. Most individuals had the reaction after the end of the therapeutic scheme.

**Table 1:** Clinical and sociodemographic characterization of study participants.

**Study** **population**	**No. of individuals**	**Age, years (Mean ±SD)**	**Sex (% male)**	**BI^**a**^ (Mean ±SD)**	**DG**^**b**^ **(%)**			**LST^**c**^**	**Time to diagnosis (months)**	**Time to reaction onset^**d**^**
					**0**	**I**	**II**			
BL/T1R	10	55.5 ± 13.2	70	3.5 ± 1.0	30	60	10	NEG	–	7 ± 1.5
BL	10	41.7 ± 22.0	81	3.9 ± 0.6	50	50	–	NEG	43	–
LL/T2R	9	47.8 ± 17.1	75	3.4 ± 2.0	41.7	50	8.3	NEG	–	17.6 ± 12.5
LL	8	28.1 ± 10.4	62	4.6 ± 1.0	87.5	12.5	–	NEG	17	–
HV^*^	15	33.5 ± 11.9	60	–	–	–	–	–	–	–

a *BI, bacteriological index*.

b *DG, disability grade*.

c *LST, lepromin skin test [NEG = negative (< 5.0 mm) and POS = positive (>5.0 mm)]*.

d *Time to onset of reaction: Measure assessed through MDT time currently recognized by WHO (6 or 12 doses)*.

* *HV, Healthy volunteers. The results were expressed as mean and standard deviation*.

We conducted a retrospective analysis in order to analyze the evolution of patients regarding the onset of reactions. One-year after blood collection, medical records were analyzed, and during this time about 39% of multibacillary patients (22% BL and 17% LL) presented one type of reactional episode during MDT.

### Downmodulation of Regulatory CD4^+^ and CD8^+^ Subpopulations in PBMC of Reactional Leprosy Patients

Anti-CD28/CD49d were used in all the culture of mononuclear cells as costimulatory molecules and to test whether Tregs influence development of leprosy reactions, we analyzed CD4^+^ and CD8^+^ Treg cells, defined as CD25^+^Foxp3^+^, in multibacillary patients, T1R, T2R and HV. Frequencies of CD4^+^ Treg subpopulations were significantly higher in BL patients when compared to T1R (*p* = 0.0001), T2R (*p* < 0.0001) and HV (*p* < 0.0001; Figure 1F). Data also demonstrated a lower frequency of CD4^+^ Treg in T2R in comparison to LL (*p* = 0.0020) and T1R (*p* = 0.0348). Similar cell frequency was observed in T1R, T2R and HV individuals. Thus, in reactional episodes, patients return to a normal CD4^+^ Treg frequency.

In regards to CD8^+^ Tregs, this cell profile decreased in BL patients during T1R genesis (*p* < 0.0001). This difference did not occur between LL and T2R individuals. This result indicates that T2R genesis is not CD8^+^ Treg dependent. However, the imbalance of this cell profile appears to be linked to T1R genesis in BL patients. Analyzing both reactional groups, T1R presents higher CD8^+^ Treg than T2R (*p* = 0.0002; Figure 1I).

CD4^+^/CD8^+^ Treg ratio was also evaluated and was shown to be <2 in T1R, BL and HV groups (Figure 1C), representing twice the amount of CD4^+^ Treg than CD8^+^. However, the CD4^+^/CD8^+^ Treg ratio was significantly higher in LL patients when compared to BL (*p* < 0.0001; Figure 1J). This high ratio is due to the lower frequency of CD8^+^ Treg than CD4^+^ Treg in LL and T2R groups. We then went on to investigate the function of these Treg subsets in patients and healthy controls.

### Regulatory T Cells Producing IL-10 Do Not Participate in Reactional Episode Pathogenesis

Given that reaction episodes in multibacillary patients are characterized by an exacerbated proinflammatory response, according to our hypothesis, this change in immune response depends on downmodulation of CD4^+^ and CD8^+^ Treg. Thus, the functional profile of Tregs producing IL-10 and TGF-β was evaluated, because these cytokines stimulate an anti-inflammatory environment, impairing Th1 and Th17 responses.

**Figure 2:**
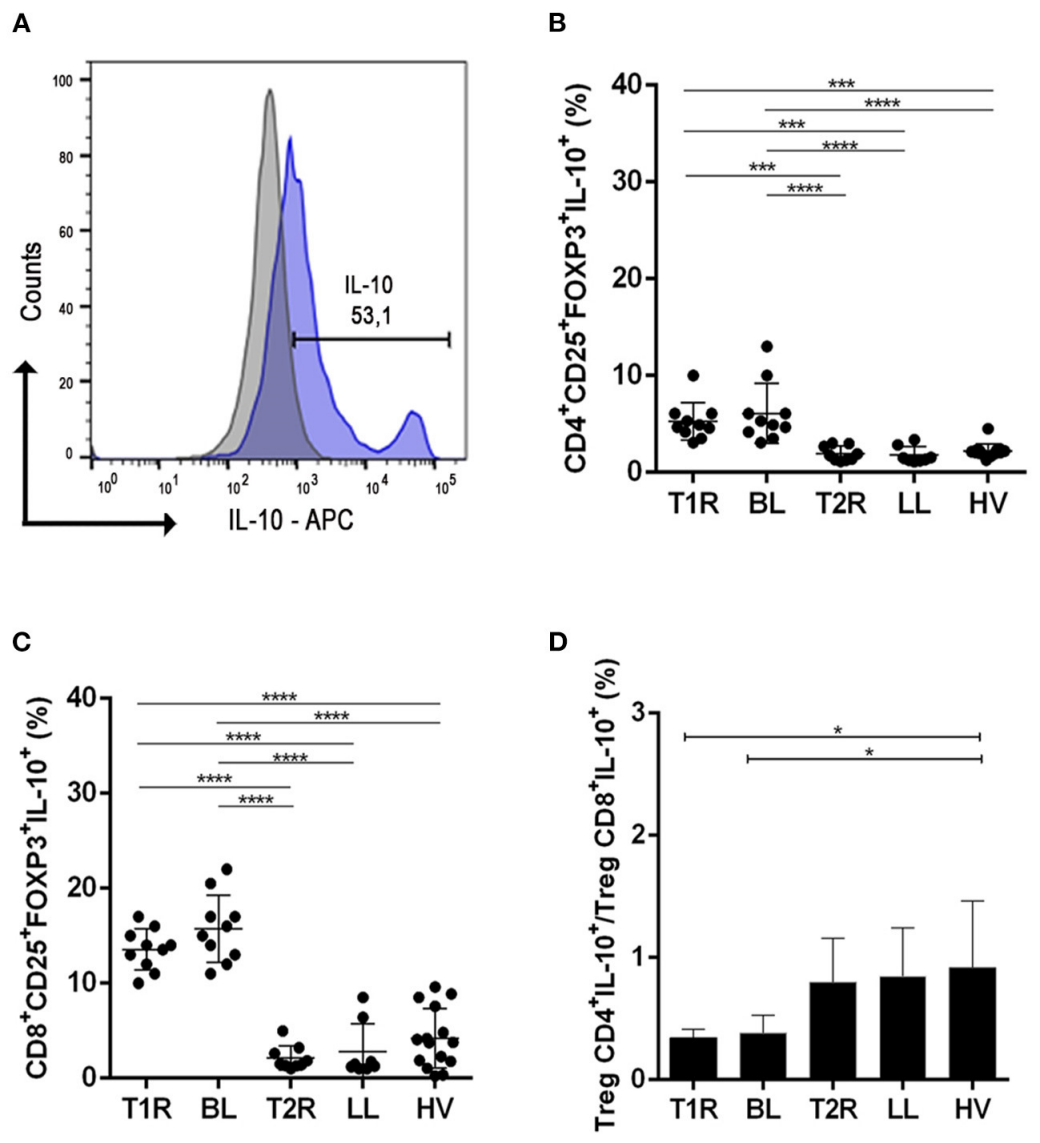
Analysis of regulatory T cells producing IL-10. Evaluation of CD4^+^ and CD8^+^ Treg frequency in blood samples in vitro with no stimuli. Representative histogram from strategy analysis, cells were gated in the regulatory T cell phenotype **(A)**; Frequency of CD4+CD25+Foxp3+IL-10+ **(B)**; CD8+CD25+Foxp3+IL-10+ **(C)**; Ratio of CD4+CD25+Foxp3+IL-10+/CD8+CD25+Foxp3+IL-10 **(D)**. Each symbol represents an individual studied according to the group; the horizontal lines represent the median. One-way Anova was used for statistical analysis where, *p ≤ 0.05, **p ≤ 0.01, ***p ≤ 0.001 and ****p ≤ 0.0001. T1R, Type 1 Reaction; BL, borderline lepromatous; T2R, Type 2 Reaction; LL, polar lepromatous patients; HV, healthy volunteers.

Peripheral frequency of IL-10 producing Treg cells was determined to be in CD4^+^CD25^+^Foxp3^+^ or CD8^+^CD25^+^Foxp3^+^ region of analysis (Figure 2A). CD4^+^CD25^+^Foxp3^+^IL-10^+^ did not present significant difference between reactional forms and non-reactional BL/LL patients. The same data was also observed for CD8^+^ IL-10+ Treg.

In T1R patients, the frequency of IL-10 producing CD4^+^ and CD8^+^ Treg was higher than in patients with T2R (*p* = 0.0006/*p* < 0.0001; Figure 2B). An elevated frequency of IL-10 producing CD4^+^ and CD8^+^ Treg was also observed in BL patients in comparison to LL ones (*p* < 0.0001/*p* < 0.0001).

Ratio analysis of CD4^+^IL-10^+^/CD8^+^IL-10^+^ Tregs was higher in HV when compared to T1R (*p* = 0.0109) and BL (*p* = 0.0158; Figure 2C). This data shows elevated CD8+IL-10+ Treg cells in T1R and BL compared to normal individuals. But between both reactional and non-reactional states, all groups had a rate below 1 (Figure 2D). This similarity shows a non-participation of CD4^+^IL-10^+^ Treg and CD8^+^IL-10^+^ Treg in genesis of reactional episodes.

### Downmodulation of CD4^+^TGF-β^+^ and CD8^+^TGF-β^+^ Cells Contribute to Pathogenesis of Leprosy Reactional Episodes

**Figure 3:**
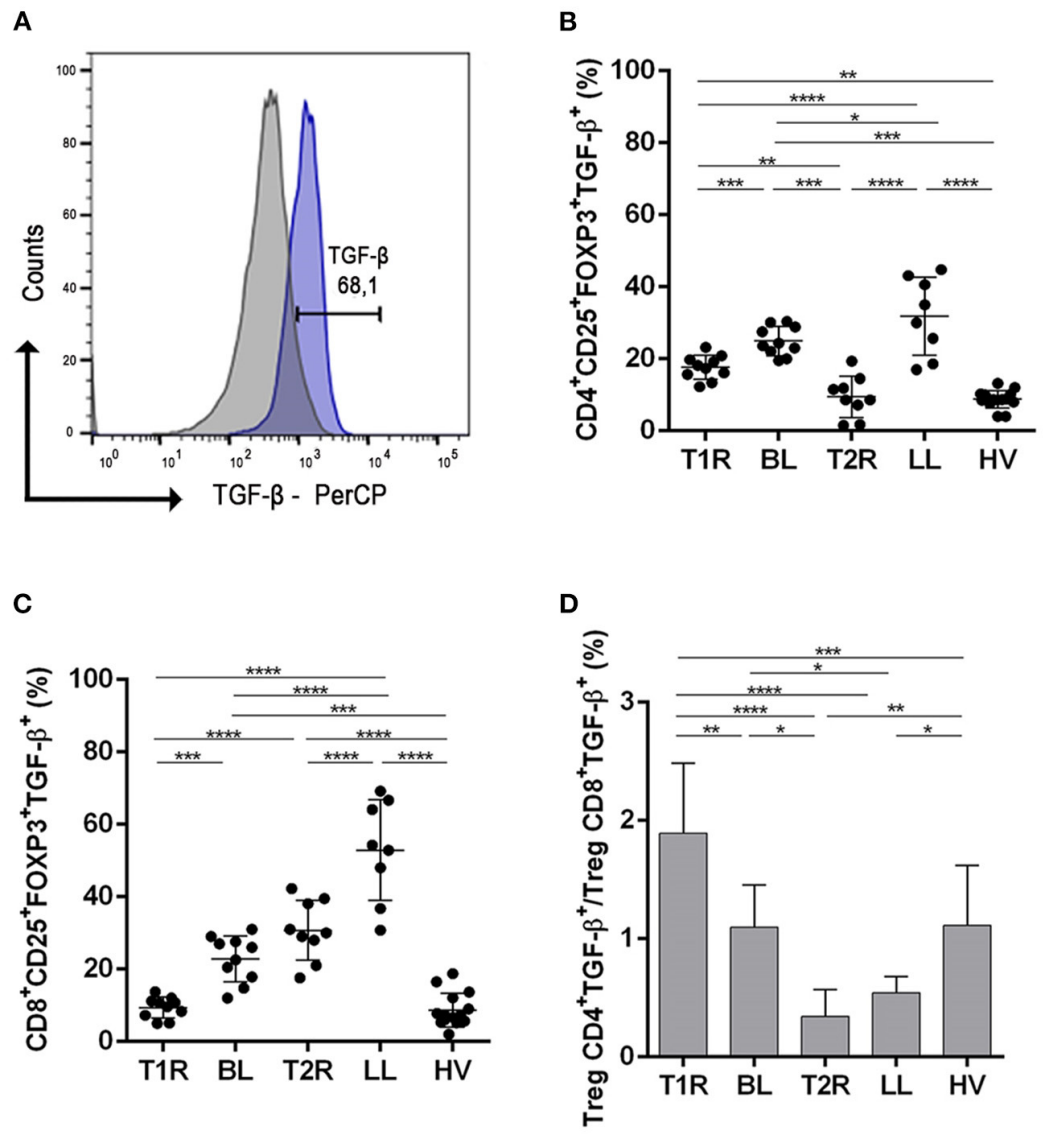
Analysis of regulatory T cells producing TGF-β. Evaluation of CD4+ and CD8+ Treg frequency in blood samples in vitro with no stimuli. Representative histogram from strategy analysis, cells were gated in regulatory T cell phenotype **(A)**; Frequency of CD4+CD25+Foxp3+TGF-β+ **(B)**; CD8+CD25+Foxp3+TGF-β+ **(C)**; Ratio of CD4+CD25+Foxp3+TGF-β+/CD8+CD25+Foxp3+TGF-β+ **(D)**. Each symbol represents an individual studied according to the group; the horizontal lines represent the median. One-way Anova was used for statistical analysis where, *p ≤ 0.05, **p ≤ 0.01, ***p ≤ 0.001 and ****p ≤ 0.0001. T1R, Type 1 Reaction; BL, borderline lepromatous; T2R, Type 2 Reaction; LL, polar lepromatous patients; HV, healthy volunteers.

Treg induce an anti-inflammatory milieu through direct contact with the target cell and immunomodulatory action through TGF-β production (Figure 3A). This cytokine is pleiotropic and participates in uncounted infectious diseases. Therefore, we evaluated the cell production of this molecule by flow cytometry.

Patients with T1R had a lower frequency of CD4^+^TGF-β^+^ Treg compared to the non-reactional BL group (*p* = 0.0132). In the T2R group, TGF-β producing CD4+ Treg was significantly lower among LL patients (*p* < 0.0001; Figure 3B). This finding revealed the participation of this cell profile in the pathogenesis of multibacillary leprosy reactions. This indicates that *Mycobacterium leprae* infection affects systemic CD4^+^TGF-β^+^ Treg frequency and downmodulation of this cell profile is related to genesis of reactions. Comparing reactional groups, T1R had a higher frequency of CD4^+^TGF-β^+^ Treg than T2R (*p* = 0.0087). Multibacillary patients also had a different TGF cell frequency and LL presented a higher CD4^+^TGF-β^+^ Treg than BL patients (*p* = 0.0215). Healthy volunteers had a significantly lower frequency of CD4^+^TGF-β^+^ Treg subsets in comparison to some groups (T1R, *p* = 0.0013; BL, *p* < 0.0001 and LL, *p* < 0.0001).

Evaluation of CD8^+^TGF-β^+^ Treg was also different between T1R and BL (*p* = 0.0132) as well as between T2R and LL (*p* = 0.0006; Figure 3C). Both reactional groups had a lower CD8^+^TGF-β^+^ Treg frequency than multibacillary leprosy, respectively. Comparing multibacillary patients, LL had a higher CD8^+^TGF-β^+^ Treg frequency than BL (*p* = 0.0215). On the other hand, when reactional episodes were compared, T2R presented higher frequency of CD8^+^TGF-β^+^ cells than T1R (*p* < 0.0001). Cell production of TGF-β also was lowest in HV in comparison to T2R (*p* < 0.0001), LL (*p* < 0.0001) and BL (*p* < 0.0001).

The ratio of CD4^+^TGF-β^+^/CD8^+^TGF-β^+^ Treg was higher in T1R when compared with T2R (*p* < 0.0001), BL (*p* = 0.0015), LL (*p* < 0.0001) and HV (*p* = 0.0007; Figure 3D), with a ratio close to or below 1. The same was observed in BL compared to T2R (*p* = 0.0024) and LL (*p* = 0.0369). The ML and PHA stimuli showed high production of TGF-β and IL-10 in all groups studied, however, no differences between them were found.

Data indicate that *Mycobacterium leprae* induces an increase of CD4^+^TGF-β^+^ and CD8^+^TGF-β^+^ Treg in multibacillary patients. This hyporesponsive milieu suffers a rupture, causing CD4^+^TGF-β^+^ and CD8^+^TGF-β^+^ Treg decrease. This new environment in addition to high proinflammatory cytokines, could trigger the genesis of both types of reactional episodes.

### Proinflammatory Cytokines Participate in T2R Episodes

To analyze the culture environment, we compared levels of proinflammatory and anti-inflammatory cytokines in PBMC supernatant. Supernatant was collected after PBMC were cultured for 72 h with or without irradiated and sonicated *M. leprae* (NIH/NIAID N01 Al-25469), phytohemagglutinin (Sigma, USA), CD28, CD49d (Biolegend, EUA) and incubated at 37°C in humidified 5% CO_2_ air.

As shown in Table 2, there was an IL-17 increase in T2R compared to non-reactional LL patients, with (*p* = 0.0173) or without *M. leprae* stimuli (*p* = 0.0177). We also observed a difference in IL-17 levels between T2R and T1R (*p* = 0.0333). IL-6 also appears to play an important role in leprosy reactions. In T2R this cytokine is increased when compared to LL patients with (*p* = 0.0056) or without *M. leprae* (*p* = 0.0025). IL-10 and IL-23 did not change significantly between reactional patients and other groups. In BL patients these cytokines may not be involved in T1R genesis.

**Table 2:** Cytokine production by PBMC, after 72 h cultures against *M. leprae* stimulus.

**Cytokynes (pg/mL)**		**T1R**	**BL**	***p* value**	**T2R**	**LL**	***p*-value**
		**Mean ±SD**	**Mean ±SD**		**Mean ±SD**	**Mean ±SD**	
IL-17	UNS	223.2 ± 41.3	176.4 ± 42.3	–	560.1 ± 299.3	207.5 ± 85.2	*
	ML	341.1 ± 80.8	196.1 ± 77.9	–	763.7 ± 316.8	295.4 ± 89.6	*
IL-23	UNS	137.7 ± 11.7	138.1 ± 13.1	–	207.9 ± 85.4	203.6 ± 81.1	–
	ML	129.8 ± 4.7	140.3 ± 9.7	–	191.7 ± 73.6	225.5 ± 74.3	–
IL-6	UNS	28.3 ± 7.7	20.3 ± 4.6	–	30.1 ± 1.8	27.1 ± 1.3	**
	ML	20.5 ± 8.7	19.7 ± 5.3	–	29.9 ± 1.1	25.9 ± 2.3	**
IFN-γ	UNS	130.8 ± 46.7	89.6 ± 14.8	–	153.6 ± 36.5	139.9 ± 33.7	–
	ML	142.5 ± 24.7	107.5 ± 34	–	147.2 ± 27.3	139.7 ± 40.9	–
IL-10	UNS	176.6 ± 10.9	157.8 ± 10.5	–	94.1 ± 39.4	137.7 ± 49.3	–
	ML	165.3 ± 30.2	160.3 ± 11.1	–	69.7 ± 24.5	88.6 ± 39.8	–

The results were expressed as mean and standard deviation. The Mann-Whitney was used for statistical analysis where, *p ≤ 0.05, **p ≤ 0.01. T1R, Type 1 reaction; BL, borderline lepromatous; T2R, Type 2 reaction; LL, polar lepromatous patients; SD, standard deviation; UNS, unstimulated; ML, M. leprae stimulus. It was analyzed BL 7, T1R 3, LL 10 and T2R 10 individuals.

## Discussion

Mechanisms involved in immunopathogenesis of reactions are not entirely understood. Our data revealed that reactions appear to be related to downmodulation of CD4^+^TGF-β^+^ and CD8^+^TGF-β^+^ Treg cells in multibacillary patients, associated to imbalance of proinflammatory cytokines. These findings bring new Treg perspectives to the pathogenesis of leprosy reactions, especially regarding the role of CD8^+^ Treg, a cell subset that has been poorly studied, but which has an important role in the clinical course of leprosy.

Most patients involved in this study were male. A similar proportion is observed in the world (1) and Brazilian epidemiological data among multibacillary patients (35). This is expected because disease impairment is related to lifestyle, work, and higher levels of testosterone found in this gender (36).

Regarding the genesis of reactional episodes, the high BI and number of lesions in LL patients are directly related to risk of initiating T2R (6). In our study, all lepromatous patients presented more than 20 widespread lesions over the body and a BI higher than 4.

Of note, anti-CD-28 and anti-CD49d antibodies were added in all the conditions of culture. The use of such molecules triggers the engagement of co-stimulatory receptors leads to recruitment of specific binding partners, such as adaptor molecules, kinases, and phosphatases, *via* recognition of a specific motif. Consequently, each co-stimulatory receptor transduces a unique pattern of signaling pathways (37, 38).

According to Sakaguchi et al. (20) and Corthay (21), regulatory T cells are identified as CD4^+^ or CD8^+^ subsets expressing CD25^+^FOXP3^+^. Therefore, characterization of this subpopulation started after duplet exclusion, viable cell determination and delimitation of lymphocyte area, followed by establishment of CD4^+^ and CD8^+^ gates. After analysis, our data revealed that negative modulation of TGF-β producing CD4^+^ and CD8^+^ Treg in multibacillary patients is related to reactional episodes. Similar results also showed the participation of CD4^+^ Treg in PBMC of T2R patients (32, 33) and in skin lesions (39).

Although our group also analyzed CTLA-4 frequency in regulatory T cells, reactional patients showed no differences when compared to multibacillary individuals. Viera e collaborators also observed that CTLA-4 does not participate as a suppressor molecule in multibacillary leprosy patients. Similar conclusions were obtained by Li and colleagues in patients with multidrug-resistant tuberculosis (27).

As in our current work, Negera and co-works showed no reduction in CD8^+^ Treg frequency in T2R, however, our study revealed that downregulation of CD8^+^TGF-β^+^ Treg participates in the onset of T1R and T2R (33). Thus, according to our hypothesis, the genesis of reactions is dependent on depletion of TGF-β producing CD4^+^ and CD8^+^ Treg. TGF-β is a cytokine capable of inhibiting macrophage activation and effector T lymphocyte action, reducing the secretion of proinflammatory cytokines, such as IFN-γ and TNF, in addition to favoring the Th17 profile shift (40, 41). Several researchers have shown TGF-β to induce apoptosis in different cells such tumors (42), embryonic cells (43), hepatocytes during alcohol abuse (44) and T lymphocytes infected by the bacillus Calmette-Guérin (BCG) vaccine provoking decreased protection against tuberculosis (45). It is possible that Treg cells may undergo apoptosis during the pathogenesis of leprosy reactions due to TGF-β.

In patients with active tuberculosis (TB), suppression of the proinflammatory response was observed before treatment. These patients had increased apoptosis of T lymphocytes in PBMC and cells in the pleural space, compared to healthy individuals (46). Also, in a coculture of PBMC from TB patients, high apoptosis of CD4^+^ T lymphocytes was revealed when compared to controls. Complementary assays show an increase in FasL on the surface of CD4^+^ T cells/m-RNA and BCL-2 reduction. Under the above conditions, TGF neutralization led to CD4 T lymphocyte survival (47). Quaresma et al. (48) demonstrated the relationship between TGF-β and caspase-3 in skin lesions of lepromatous patients. Thus, our data indicates that exacerbated production of TGF-β may be related to Treg apoptosis during onset of reactional episodes.

Higher frequency of TGF-β producing cells in multibacillary patients over time, could thus trigger apoptosis of Treg in an autocrine or paracrine manner, favoring performance of cells and cytokines with a proinflammatory profile and inducing reactions. Furthermore, apoptosis of these TGF-β producing Treg cells possibly appears to be linked to an increased frequency of a T lymphocyte proinflammatory environment that is widely recognized in the pathogenesis of these reaction episodes.

Previous work by our group had already shown a high frequency of TBX21 as well as an increase in IFN-γ producing CD4^+^ T cells in the genesis of T1R in borderline lepromatous patients (17). On the other hand, in T2R we found an increased in CD8^+^ TNF producing cells (18). Thus, after apoptosis of TGF-β^+^ Treg an increase in CD4^+^IFN-γ^+^ and CD8^+^TNF^+^ cells could occur, triggering T1R and T2R, respectively.

In addition, the present work also revealed the participation of IL-17 and IL-6 in the genesis of T2R in lepromatous patients. According to Saini and colleagues, the high *in vitro* production of IL-6 occurs in monocytes and granulocytes in T2R (32), and after treatment with prednisone there is a significant reduction of this cytokine (49). IL-6 and IL-17 are closely related to TGF-β. In synergy with IL-6, TGF-β stimulates the polarization of CD4^+^ T lymphocytes to the Th17 profile, a subpopulation with proinflammatory action through the production of IL-17, IL-22, and IL-23 (50, 51). Increase in IL-17 producing T lymphocytes had already been observed in the pathogenesis of T2R in Ethiopian multibacillary patients (33). Negera also observed a reduction of the Th17 subpopulation after starting treatment with prednisone. According to our data, after Treg apoptosis there is an increase in IL-17, possibly triggering a proinflammatory environment in T2R. Moreover, pathogenesis of T2R appears to be independent of IL-23 presence, since this cytokine was not altered in the patients studied. This same data was also observed through RT-PCR analysis of skin lesions (25).

IFN-γ positive modulation may participate in some way in both Type 1 and 2 reactions, along with the negative modulation of IL-10 in T2R. These data appear to show a breakdown in the balance between an anti-inflammatory environment, maintained by Treg in multibacillary patients, and the beginning of reactions marked by a proinflammatory profile, with the involvement of the Th17 shift and IL17 production. This phenomenon can occur in T1R patients, but other mechanisms and mediators can participate in triggering the reaction.

Interleukin-10 producing CD4^+^ and CD8^+^ Treg and the presence of IL-10 in culture supernatants does not appear to be crucial for the maintenance of the hyporesponsive state observed in multibacillary patients under study conditions. Saini and collaborators observed CD4^+^TGF-β^+^ Treg in multibacillary patients (52). This frequency was also observed in multibacillary patients when compared with paucibacillary ones (32). Saini data corroborate our findings and suggest that IL-10 producing CD4^+^ and CD8^+^ Treg may not be crucial for maintaining the *Mycobacterium leprae* response in multibacillary patients.

Future experimental studies could demonstrate the mechanism proposed in this work such as adding neutralizing antibodies for TGF-β in PBMC cultures of multibacillary and reactional patients, as well as apoptosis assay analysis. In conclusion, our study showed the participation of TGF-β producing CD4^+^ and CD8^+^ Treg in the maintenance of a hyporesponsive profile in multibacillary leprosy patients. Thus, apoptosis of Treg subpopulations could be related to overproduction of TGF-β, promoting an imbalance of this cell profile and leading to genesis of T1R and T2R episodes. Finally, these conditions are influenced by higher synthesis of IL-17 and IL-6 in T2R but not in T1R pathogenesis.

## Data Availability Statement

The original contributions presented in the study are included in the article/supplementary materials, further inquiries can be directed to the corresponding author.

## Ethics Statement

The studies involving human participants were reviewed and approved by Ethics Committee of the Oswaldo Cruz Foundation/FIOCRUZ (protocol number 518/09). The patients/participants provided their written informed consent to participate in this study.

## Author Contributions

DE: conceptualization, funding acquisition, review, and editing the text. KG, PL, LN, JL, and MP: performed the experiments. IA: analyzed data of flow cytometric experiments. KG, PL, and DE: analyzed the data and writing the manuscript. All authors contributed to the article and approved the submitted version.

## Funding

This investigation received financial support from PAEF (in Portuguese: Projeto de Ações Estratégicas para Desenvolvimento e Fortalecimento dos Laboratórios Credenciados e das Áreas de Apoio à Pesquisa do IOC). KG is postgraduate student sponsored by FIOCRUZ/CNPq (in Portuguese: Conselho Nacional de Pesquisa e Desenvolvimento Tecnológico, number 18.08.38.140). The funders had no role in the study design, data collection and analysis, decision to publish, or preparation of the manuscript.

## Conflict of Interest

The authors declare that the research was conducted in the absence of any commercial or financial relationships that could be construed as a potential conflict of interest.

## Publisher's Note

All claims expressed in this article are solely those of the authors and do not necessarily represent those of their affiliated organizations, or those of the publisher, the editors and the reviewers. Any product that may be evaluated in this article, or claim that may be made by its manufacturer, is not guaranteed or endorsed by the publisher.
